# EpCAM regulates hair cell development and regeneration in the zebrafish lateral line

**DOI:** 10.17912/micropub.biology.001219

**Published:** 2024-05-08

**Authors:** Andrew J. Bufalo, Madison M. Vanderbeck, Jodie Schmitt, Hillary F. McGraw

**Affiliations:** 1 Division of Biology and Biomedical Systems, University of Missouri–Kansas City, Kansas City, Missouri, United States; 2 Division of Biological and Biomedical Systems, University of Missouri–Kansas City, Kansas City, Missouri, United States

## Abstract

The zebrafish lateral line mechanosensory system shares considerable morphological and molecular similarities with the inner ear. In particular, mechanosensory hair cells are responsible for transducing sensory stimuli in both structures. The epithelia cell adhesion molecule (EpCAM) is expressed in the cells of the inner ear of mammals and in the lateral lines system of fish. EpCAM regulates the many cellular functions including adhesion, migration, proliferation, and differentiation. In this study, we use the *epcam ^jh79^* mutant zebrafish line to determine that EpCAM function is required for proper development and regeneration of posterior lateral line hair cells.

**Figure 1.**
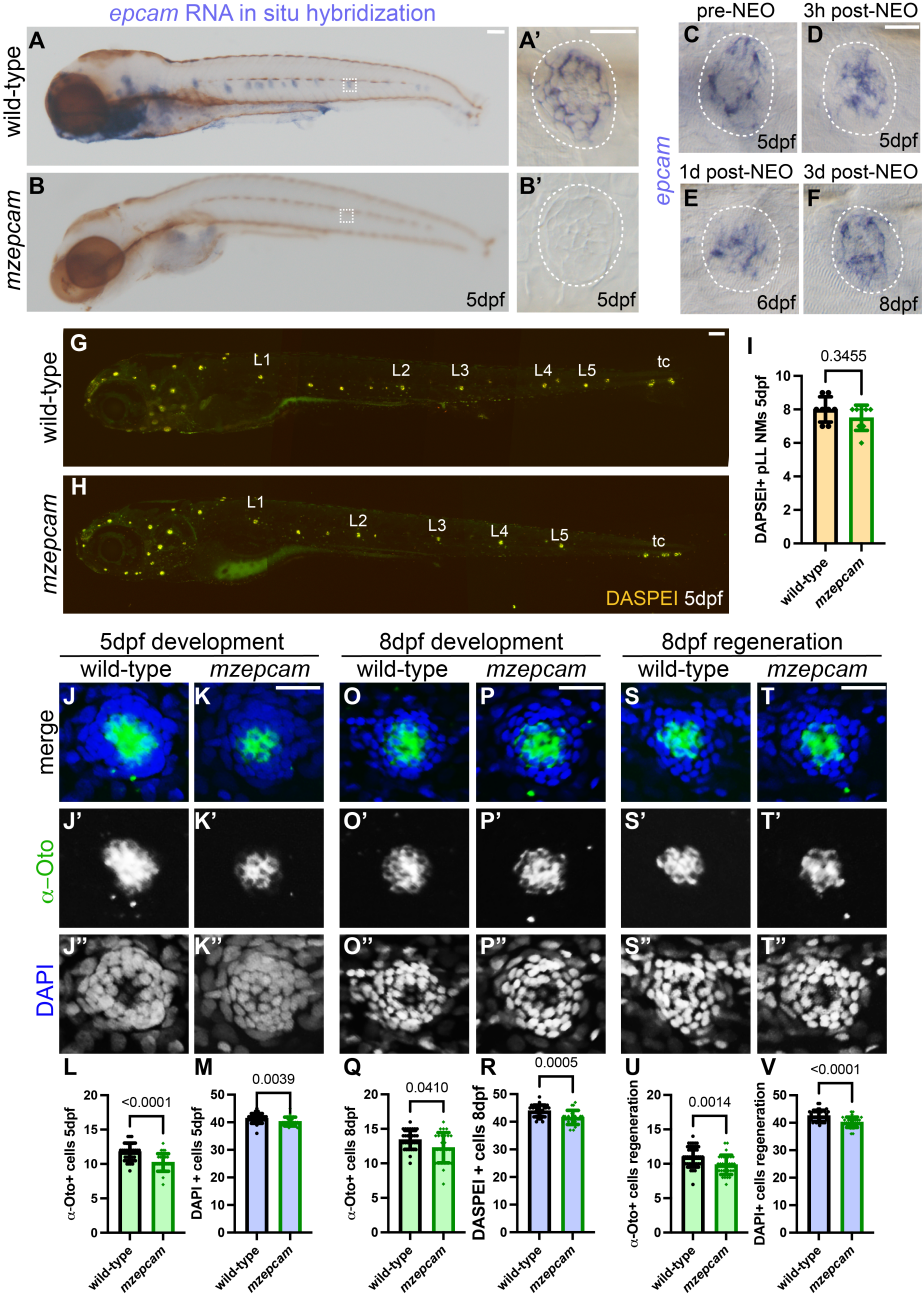
EpCAM regulates posterior lateral line neuromast development and regeneration. (A-B) Whole mount images of 5pdf wild-type and *mzepcam* mutant zebrafish larvae showing expression of *epcam* mRNA by in situ hybridization. Scale bar=100μm. Dashed squares show area of individual neuromasts in (A’-B’). Scale bar=20μm. (C-F) RNA in situ hybridization of *epcam* at 5dpf pre-NEO exposure, 3-hours post-NEO exposure, 1-day post-NEO exposure at 6dpf, and 3-days post-NEO exposure at 8dpf. Scale bar=20μm. (G-H) Live confocal projections of 5pdf wild-type and *mzepcam* mutant larvae showing hair cells labeled with DASPEI in the primary posterior lateral line neuromasts (L1-L5) and terminal cluster (tc) neuromasts. Scale bar=100μm. (I) Quantification of DASPEI-labeled posterior lateral line neuromasts in 5pdf wild-type and *mzepcam* mutant zebrafish. n=8 larvae per condition. Data presented as ± S.D. Mann-Whitney test, significance at p<0.05. (J-K’’) Confocal projections of neuromasts in 5pdf wild-type and *mzepcam* mutant larvae with hair cells labeled with a-Oto antibody and nuclei labeled with DAPI. Scale bar=20μm. (L-M) Quantification of α-Oto-labeled hair cells and DAPI total cells in neuromasts of 5pdf wild-type and *mzepcam* mutant larvae. n=29 neuromasts in 9 wild-type and n=27 neuromasts in 10 *mzepcam* mutant larvae. Data presented as ± S.D. Mann-Whitney test. Data presented as ± S.D. Mann-Whitney test, significance at p<0.05. (O-P’’) Confocal projections of neuromasts in 8pdf wild-type and *mzepcam* mutant larvae with hair cells labeled with α-Oto antibody and nuclei labeled with DAPI. Scale bar=20μm. (Q-R) Quantification of α-Oto-labeled hair cells and DAPI total cells in neuromasts of 8pdf wild-type and *mzepcam* mutant larvae. n=32 neuromasts in 10 wild-type and n=27 neuromasts in 10 *mzepcam* mutant larvae. Data presented as ± S.D. Mann-Whitney test. Data presented as ± S.D. Mann-Whitney test, significance at p<0.05. (S-T’’) Confocal projections of neuromasts in 8pdf following hair cell ablation with NEO at 5dpf in wild-type and *mzepcam* mutant larvae with hair cells labeled with α-Oto antibody and nuclei labeled with DAPI. Scale bar=20μm. (U-V) Quantification of regenerated α-Oto-labeled hair cells and DAPI total cells in neuromasts of 8pdf wild-type and *mzepcam* mutant larvae. n=33 neuromasts in 11 wild-type and n=32 neuromasts in 11 *mzepcam* mutant larvae. Data presented as ± S.D. Mann-Whitney test. Data presented as ± S.D. Mann-Whitney test, significance at p<0.05. Abbreviations: days post fertilization (dpf), neomycin (NEO), standard deviation (S.D.), α-Otoferlin antibody (α-Oto).

## Description

The sensations of hearing, balance, and in the case of aquatic vertebrates, water current are transduced by specialized mechanosensory hair cells (Pickett & Raible, 2019) . In mammals, damage to hair cells results in irreversible sensory loss (White, 2020) . In contrast, non-mammalian vertebrates are able to regenerate damaged hair cells (Denans, Baek, & Piotrowski, 2019; Kniss, Jiang, & Piotrowski, 2016; Thomas, Cruz, Hailey, & Raible, 2015) . In particular, the lateral line mechanosensory system of the zebrafish, *Danio rerio* , has immerged as an excellent model to study the biology of hair cell development and regeneration (Kniss et al., 2016; Thomas et al., 2015) . The lateral line is composed of sensory organs, called neuromasts, which are arrayed along the surface of the body. Within these neuromasts are mechanosensory hair cells and surrounding support cells, which act as stem cells to replace hair cells following damage (Kniss et al., 2016) . Understanding the molecular mechanisms that regulate hair cell regeneration in the zebrafish lateral line could provide clues for potential therapeutic regrowth of hair cells in the human inner ear. In this study, we examine the role of the epithelial cell adhesion molecule (EpCAM) in lateral line development and regeneration. EpCAM is a membrane glycoprotein, which regulates many cellular functions including adhesion, proliferation, stem cell maintenance and differentiation, migration, and invasion of cancer cells (Fagotto, 2020; Fagotto & Aslemarz, 2020; Gires, Pan, Schinke, Canis, & Baeuerle, 2020; Liu et al., 2022) . EpCAM is expressed in the mammalian inner ear and may play a role in specifying hair cell progenitors (Roccio et al., 2018) . In this study, we examine the function of EpCAM in the development and regeneration of zebrafish posterior lateral line neuromast cells.

To investigate the role of EpCAM in the development of posterior lateral line neuromasts, we used homozygous maternal zygotic *epcam ^jh79^* ( *mzepcam* ) (Edelman et al., 2021) mutant zebrafish larvae and compared them to AB wild-type control larvae. We examined *epcam* expression using RNA in situ hybridization at 5 days post fertilization (dpf), when the lateral line has reached initial functional maturity (Thomas et al., 2015) , and found expression in the neuromasts of the posterior lateral line of wild-type larvae ( Fig. 1A,A ’), but no expression in *mzepcam* larvae ( Fig. 1B,B ’). This finding agrees with previous reports that the *mzepcam* mutants show nonsense mediated mRNA decay (Edelman et al., 2021) . As *epcam* expression is present under homeostatic conditions in lateral line neuromasts, we next asked if expression was altered following hair cell ablation and during regeneration. We used exposure to the aminoglycoside antibiotic neomycin (NEO) (Harris et al., 2003; Owens et al., 2008) to destroy lateral line hair cells in wild-type larvae and assessed *epcam* expression in neuromasts at 5dpf pre-NEO exposure ( Fig. 1C ), 3-hours post-NEO exposure ( Fig. 1D ), 1-day post-NEO exposure ( Fig. 1E ), and 3-days post-NEO exposure ( Fig. 1F ) when regeneration is functionally complete. We found that *epcam* mRNA expression is maintained in neuromasts during the course of hair cell regeneration.

Previous research on the function of EpCAM in the zebrafish lateral line suggests that knocking down function with antisense morpholino oligonucleotides (MO) against *epcam* results in delayed and disrupted formation (Neelathi, Dalle Nogare, & Chitnis, 2018; Villablanca et al., 2006) . To determine if EpCAM is required for formation of posterior lateral line, we compared live wild-type ( Fig. 1G ) and *mzepcam* (Fig.1H) larvae at 5dpf in which hair cells have been labeled with the vital dye DASPEI (Harris et al., 2003) and found that primary neuromast numbers are not significantly different between control and mutant larvae ( Fig. 1I ). These results suggest that EpCAM function is not required for neuromast number in the posterior lateral line.

We next asked if EpCAM is required for hair cell development in primary posterior lateral line neuromasts. We used an α-Otoferlin antibody (α-Oto) to specifically label hair cells and DAPI to label nuclei in the posterior lateral line neuromasts of 5dpf wild-type ( Fig. 1J -J’’) and *mzepcam* (Fig.1K-K’’) larvae and found a significant reduction in hair cells ( Fig. 1L ) and total DAPI+ cells ( Fig. 1M ) in mutant larvae. To determine if the decrease in cell numbers is due to delay, we also examined neuromasts at 8dpf and once again found the *mzepcam* mutant larvae have fewer hair cells and total cells in their neuromasts ( Fig. 1P -P’’, Q,R) as compared to wild-type controls ( Fig. 1O -O’’, Q,R). These data suggest that EpCAM function is required for the proper development of the full complement of hair cells and total cells in posterior lateral line neuromasts.

Finally, we asked if there is a change in the ability of hair cells to regenerate following damage in the absence of EpCAM function. We ablated hair cells at 5dpf using exposure to NEO in wild-type and *mzepcam* mutant larvae and then allowed them to regenerate for 72-hours and examined α-Oto-labeled hair cells and DAPI-labeled nuclei at 8dpf. We found that hair cells regenerated in our wild-type ( Fig. 1S -S’’) and *mzepcam* mutant neuromasts, but overall, there were fewer hair cells and total cells in mutant larva as compared to controls ( Fig. 1U,V ). Together, these results suggest that EpCAM function regulates the development and regeneration of posterior lateral line neuromast cells.

In conclusion, this work shows that *epcam* is expressed in the developing and regenerating posterior lateral line neuromasts of zebrafish larvae. In the absence of EpCAM function, the full complement of neuromasts form by 5dpf, suggesting that EpCAM may be dispensable for this process. When we specifically examined hair development and regeneration in neuromasts, we find the loss of EpCAM function results in reduced cell numbers. These results suggest that EpCAM plays a role in regulating the formation and regrowth of cells within posterior lateral line neuromasts. Future work is needed to determine the mechanism of EpCAM activity. In other tissues and model systems, EpCAM interacts with canonical Wnt signaling pathway (Gires et al., 2020) . Previous work has shown that Wnt signaling regulates the development and regeneration of the posterior lateral line in zebrafish (Megerson et al., 2024; Romero-Carvajal et al., 2015) and might interact with EpCAM in neuromasts. EpCAM regulates the development of the ear in mammals and in fish (Edelman et al., 2021; Han et al., 2011; Roccio et al., 2018; Ueda et al., 2023) ; moreover, future work is required to determine if it might be a target for clinical intervention in hearing loss.

## Methods

**Zebrafish lines and maintenance**

The following zebrafish lines were used: wild-type*AB (ZIRC;http://zebrafish.org) and *epcam ^jh79^* (Edelman et al., 2021) *.* Adult zebrafish were maintained in a facility with 14 hour-light/10 hour-dark and water temperature at 28°C. Larvae were kept at 28°C and staged according to standard protocols (Kimmel, Ballard, Kimmel, Ullmann, & Schilling, 1995) . This study used larvae at stages between 5-8dpf. *epcam ^jh79^* fish were maintained as homozygous mutant adults and progeny used for experiments were maternal zygotic homozygous mutants ( *mzepcam* ). Experimental larvae were kept in E3 embryo medium (14.97 mM NaCl, 500 μM KCl, 42 μM Na _2_ HPO _4_ , 150 μM KH _2_ PO _4_ , 1 mM CaCl _2_ dihydrate, 1 mM MgSO _4_ , 0.714 mM NaHCO _3_ , pH 7.2). For all experiments, larvae were treated with tricaine (Syndel) prior to fixation in 4% paraformaldehyde/PBS (Thermo Fisher). All work was performed in accordance with the McGraw laboratory protocol #45344 approved by the UMKC IACUC committee.

**RNA in situ hybridization**

RNA in situ hybridization was carried out using established protocols (Thisse & Thisse, 2008) , modified with a 5-minute Proteinase K (Thermo Fisher) treatment to preserve neuromast integrity. Following coloration, the larvae were cleared in methanol and stored in 50% glycerol/PBS for imaging *.* Antisense probe for *epcam* was generated using a PCR-based protocol (Logel, Dill, & Leonard, 1992) in which the cDNA of interest is amplified by PCR with a reverse primer containing a T7 RNA binding sequence (CCAAGCTTCTAATACGACTCACTATAGGGAGA). Following PCR amplification of probe template, antisense RNA probes were generated with T7 RNA polymerase and digoxygenin RNA labeling mix.

**Immunohistochemistry and DASPEI labeling.**

Whole mount immunolabeling was performed using established protocols (Ungos, Karlstrom, & Raible, 2003) . The α-Otoferlin primary antibody (α-Oto; mouse monoclonal, 1:200, DSHB, University of Iowa) was diluted in blocking solution and larvae were incubated overnight at 4°C, then washed in 1x PBS-0.1% Triton-X 100 and incubated overnight in goat anti-mouse Alexa-647 (1:1000, Invitrogen) secondary antibody diluted to 1:1000. Larvae were stored in 50% glycerol/PBS for imaging. For live imaging of neuromasts, 5dpf larvae were incubated in 0.005% (2-(4-(Dimethylamino)styryl)-N-ethylpyridinium iodide) (DASPEI) in E3 embryo medium for 15 minutes and then rinsed in clean E3 (Harris et al., 2003) . Larvae were anesthetized in tricaine for imaging.

**Neomycin exposure and regeneration**

For hair cell ablation, 5 dpf larvae were incubated in 400 μM neomycin (NEO, Millipore-Sigma) in E3 embryo medium for 0.5 hours and then washed 3x in fresh E3 embryo medium (Harris et al., 2003) . In experiments analyzing complete regeneration, larvae were collected 3 days following NEO exposure. For RNA in situ hybridization, larvae were collected 3-hours, 1-day, or 3-days post NEO exposure.

**Image collection**

For imaging of RNA in situ hybridization and immunohistochemistry, processed larvae were placed in 50% glycerol/PBS and mounted on slides. Images were collected using a Zeiss Imager.D2 compound microscope with an Axiocan 506 color camera and Zen software. Fluorescent images were collected using a Zeiss 510 meta confocal microscope using Zen 2009 software. Images were processed using Fiji software (Schindelin et al., 2012) , and brightness and contrast were adjusted using Photoshop (Adobe). Neuromasts L2-L4 were analyzed for each fish and cells were manually counted under blinded conditions.

**Quantification and statistical analysis**

All statistical analysis was carried out using Graphpad Prism 10 (GraphPad Prism version 10.0.0 for Mac, GraphPad Software, San Diego, California USA, www.graphpad.com). A Mann-Whitney nonparametric test was used for all pair-wise comparisons. Significance was set at p<0.05. All data is presented as ± standard deviation (S.D.).

## Reagents

**Table:** 

REAGENT or RESOURCE	SOURCE	IDENTIFIER
Antibodies		
Mouse monoclonal anti-Otoferlin antibody (HCS-1)	DSHB	Cat.#HCS-1; RRID:AB_10804296
Alexa fluor-674 goat anti-mouse antibody	Thermo Fisher	Cat.# A-21236; RRID:AB_2535805
Anti-Digoxigenin-AP, Fab Fragments	Millipore Sigma	Cat.# 11093274910; RRID:AB_2734716
Chemicals, peptides, and recombinant proteins		
(2-(4-(Dimethylamino)styryl)-N-ethylpyridinium iodide) (DASPEI)	BIOTIUM	70018
DAPI	Thermo Fisher	D1306
Neomycin sulphate (NEO)	Millipore Sigma	N0400000
Digoxygenin RNA labeling mix	Millipore Sigma	11277073901
Experimental models: Organisms/strains		
Zebrafish: Wildtype *AB	ZIRC http://zebrafish.org	ZFIN:ZDB-GENO-960809-7
Zebrafish: *epcam ^jh79^*	(Edelman et al., 2021)	ZFIN:ZDB-ALT220225-4
Oligonucleotides		
*epcam* in situ hybridization probe template primers Forward:5’GGTTCCCAAGTGCTTCCTCA3’ Reverse:5’CCAAGCTTCTAATACGACTCACTATAGGGAGACAGCACAACTGCCCCAATTT3’	IDT	ZFIN:ZDB-GENE-040426-2209
Software and algorithms		
FIJI	(Schindelin et al., 2012)	https://imagej.nih.gov/ij/
PRISM	GraphPad	www.graphpad.co
